# Combination Treatment with an Antibody–Drug Conjugate (A1mcMMAF) Targeting the Oncofetal Glycoprotein 5T4 and Carboplatin Improves Survival in a Xenograft Model of Ovarian Cancer

**DOI:** 10.1007/s11523-019-00650-8

**Published:** 2019-07-22

**Authors:** Y. Louise Wan, Puja Sapra, James Bolton, Jia Xin Chua, Lindy G. Durrant, Peter L. Stern

**Affiliations:** 1 Division of Cancer Sciences, School of Medical Sciences, Faculty of Biology, Medicine and Health, The University of Manchester, 5th Floor Research, St Mary’s Hospital, Oxford Road, Manchester, M13 9WL UK; 2 Oncology Research and Development, Pfizer Inc., 401 N. Middletown Road, Pearl River, NY 10954 USA; 3 Department of Histopathology, Manchester University NHS Foundation Trust, Oxford Road, Manchester, M13 9WL UK; 4 Academic Clinical Oncology, The University of Nottingham, City Hospital Campus, Hucknall Road, Nottingham, NG5 1PB UK; 5 Manchester Cancer Research Centre, Division of Cancer Sciences, School of Medical Sciences, Faculty of Biology, Medicine and Health, University of Manchester, Wilmslow Road, Manchester, M20 4BX UK

## Abstract

**Background:**

Recurrence occurs in over 75% of women with epithelial ovarian cancer despite optimal treatment. Selectively killing tumour cells thought to initiate relapse using an antibody–drug conjugate could prolong progression-free survival and offer an improved side-effect profile. A1mcMMAF is an antibody–drug conjugate designed to target cells expressing the tumour-associated antigen 5T4. It has shown to be efficacious in various cell line models and have a greater impact when combined with routine chemotherapeutic regimes.

**Objectives:**

This study aims to explore the potential for the use of a 5T4 antibody–drug conjugate in women with ovarian cancer both as a monotherapy and in combination with platinum-based chemotherapy.

**Methods:**

Immunohistochemical analysis was used to assess 5T4 expression in tumours from patients with ovarian cancer. Effectiveness of A1mcMMAF therapy as a single agent and in combination with carboplatin was assessed in vitro in the ovarian cancer cell line SKOV3 and confirmed in vivo using a serial bioluminescence assay in a SKOV3 xenograft model of ovarian cancer.

**Results:**

5T4 is confirmed as suitably expressed in epithelial ovarian cancers prior to adjuvant therapy and is an independent predictor of poor survival. A1mcMMAF showed specific activity, both in vitro and in vivo, against SKOV3 ovarian cancer cells. When used in combination with carboplatin, in vivo tumour growth was inhibited resulting in prolonged survival in a SKOV3 xenograft model.

**Conclusions:**

These data support further investigation of A1mcMMAF in combination with platinum-based chemotherapy in ovarian and other cancer treatments.

**Electronic supplementary material:**

The online version of this article (10.1007/s11523-019-00650-8) contains supplementary material, which is available to authorized users.

## Key Points

**Table:** 

5T4 is widely expressed in epithelial ovarian cancers and is associated with poor prognosis.
5T4 expression has been associated with cancer spread and relapse and therefore targeting cells expressing this oncofetal protein may limit recurrence and extend survival.
The 5T4-specific antibody–drug conjugate, A1mcMMAF, delays progression and extends survival when used as a single agent in a model of ovarian cancer. This effect is enhanced when A1mcMMAF is used in combination with carboplatin chemotherapy.

## Introduction

Epithelial ovarian cancer (EOC) is one of the most common gynaecologic malignancies, with 50% of all cases occurring in women aged older than 65 years. It is the eighth most frequent cause of cancer death in women [1]. The most common subtype of EOC, high-grade serous cancer (70%), is characterised by high levels of genomic instability resulting in frequent genomic aberrations and morphological heterogeneity. This makes the identification of key driver mutations and the development of therapeutics against such mutations challenging [2, 3]. Whilst this genomic instability initially sensitises high-grade serous EOCs to DNA-damaging agents and sensitisers, such as carboplatin and poly-ADP ribose polymerase inhibitors [4–6], ultimately, the inherent molecular heterogeneity and plasticity of EOC cells mean that 80–90% of women originally treated with these agents will develop resistance [7].

Antibody–drug conjugates (ADCs) are an emerging class of anticancer drug that combine the selectivity of a monoclonal antibody with the cytotoxic potency of established chemotherapeutics. Selectively targeting tumour cells thought to initiate relapse could prolong progression-free survival whilst limiting potential off-target side effects [8]. The 5T4 oncofetal glycoprotein exhibits several properties that make it a highly attractive target antigen in this regard. 5T4 is rarely expressed by normal adult tissues [9, 10], but is detected in a high proportion of primary and metastatic cancers, including EOC [11–13]. Mechanistically, 5T4 expression has been shown to associate with several processes that are important in the spread of cancer cells and relapse [14]. These include epithelial-mesenchymal transition [15, 16], the directional movement of cells through potentiation of CXCL12/CXCR4 chemotaxis [17, 18] and inhibition of canonical Wnt/beta catenin and concurrent potentiation of non-canonical pathway signalling [19]. Consistent with this role in tumour development and spread, 5T4 expression in colorectal, gastric and all subtypes of ovarian cancer has been correlated with a poorer clinical outcome [11, 20–24].

These characteristics have underpinned the clinical development of various immunotherapies [12, 14] including a number of ADCs [25–27]. A1mcMMAF is a 5T4 humanised monoclonal antibody (A1) chemically linked by a non-cleavable maleimidocaproyl linker to deliver a microtubule-disrupting agent, monomethyl auristatin F (MMAF). A1mcMMAF has been shown to be rapidly internalised on binding of the antibody portion and to have potent in vivo activity in a variety of tumour models [26] leading to the initiation of a phase I dose-escalation study [25]. Recent work has identified that combining a 5T4 ADC with various other chemotherapeutics enhances its effects on survival [28, 29]. These data support further investigation of A1mcMMAF in combination with chemotherapy in other cancers.

## Materials and Methods

### Ethics Approval and Consent to Participate

Animal work was performed in accordance with the UK Animal Scientific Procedures Act 1986 and was covered by both project (PPL 40/3200) and personal licences that were issued by the Home Office and reviewed by the Manchester Institute for Cancer Research Ethics Committee. Patient samples and data were collected and analysed with the approval of the Derby Royal Hospital Ethics Committee and the Nottingham Research Ethics Committee (0205/495).

### Patient Samples

The tissue microarray comprising 360 consecutive cases of EOC collated between 1984 and 1997 and corresponding validated clinical data have been previously described [30]. Clinicopathological variables for the cohort are outlined in Table 1.

**Table 1:** Clinicopathological variables of the analysed cohort

Variable	Categories	Frequency of the analysed cohort (%), *n *= 271	Frequency of total cohort (%), *n* = 360
SEER^a^ age category	< 30 years at diagnosis	1 (< 1)	2 (< 1)
	30–60 years at diagnosis	107 (39)	143 (40)
	> 60 years at diagnosis	160 (59)	212 (59)
	Unknown	3 (1)	3 (< 1)
Macroscopic residual disease	Absent	109 (40)	143 (40)
	Present	152 (56)	201 (56)
	Unknown	10 (6)	16 (4)
FIGO stage	I	77 (28)	95 (26)
	II	29 (11)	38 (11)
	III	129 (48)	175 (49)
	IV	28 (10)	40 (11)
	Unknown	8 (3)	12 (3)
Histological type	Serous carcinoma	128 (47)	178 (49)
	Mucinous cystoadenocarcinoma	27 (10)	35 (10)
	Endometrioid	37 (14)	42 (12)
	Clear cell	21 (8)	25 (7)
	Undifferentiated	42 (15)	54 (15)
	Others	16 (6)	26 (7)
Serous tumour grade	High	119 (44)	160 (44)
	Low	14 (5)	18 (5)
Tumour grade of all other tumours	Well differentiated (3)	77 (28)	100 (28)
	Moderately differentiated (2)	32 (11)	39 (11)
	Poorly differentiated (1)	17 (6)	20 (6)
	Unknown	17 (6)	23 (6)
Adjuvant therapy	No	76 (28)	101 (28)
	Yes	187 (69)	249 (69)
	Unknown	8 (3)	10 (3)

*FIGO* International Federation of Gynecology and Obstetrics, *SEER* Surveillance, Epidemiology, and End Results

^a^SEER standardised age categories

### Immunohistochemistry

The automated Ventana BenchMark ULTRA IHC⁄ISH Staining Module (Roche, Indianapolis, IN, USA) was used together with the Ultraview 3,3′ diaminobenzidine version 3 detection system (Roche) to label the sections for 5T4. In brief, 4-µm tissue sections were deparaffinised and a 64-min heat-induced antigen retrieval protocol was carried out using a TRIS–ethylenediamine tetraacetic acid–boric acid pH 8.4 buffer (Cell Conditioner 1; Roche). Sections were incubated with ultraviolet inhibitor blocking solution (Roche) and then labelled with a monoclonal rabbit antibody to 5T4 (ab134162) (Abcam, Cambridge, UK) [31] at a 1:1000 dilution or normal rabbit serum (Sigma-Aldrich, St Louis, MO, USA), as a negative control, for 40 min at room temperature. This was followed by incubation with a horseradish peroxidase-linked secondary antibody (8 min) (Roche), 3,3′ diaminobenzidine chromogen and substrate (8 min) (Roche), and a copper enhancer for 4 min (Roche). Counterstain (haematoxylin II) was then applied. Human placental sections, kindly donated by the Manchester Biomedical Research Centre Biobank, were used as positive and negative tissue controls. Staining was determined as appropriate if placental syncytiotrophoblast cells stained positive for 5T4 and stromal cells stained negative for 5T4 [32, 33].

Two independent observers (YLW and JB) assessed the percentage of the core containing the tumour. Cores containing less than 10% tumour were excluded from analysis. Staining intensity of the malignant epithelium in the remaining cores was assessed and assigned a score of between 0 and 3; with high expression equating to a score of 2 or more and low expression a score of 1 or less. Observers were blind to clinical and pathological parameters. Reporting recommendations for Tumor Marker Prognostic Studies (REMARK) criteria were followed [34].

### Generation of 5T4 Knockout SKOV3 Cell Lines

**Fig. 1:**
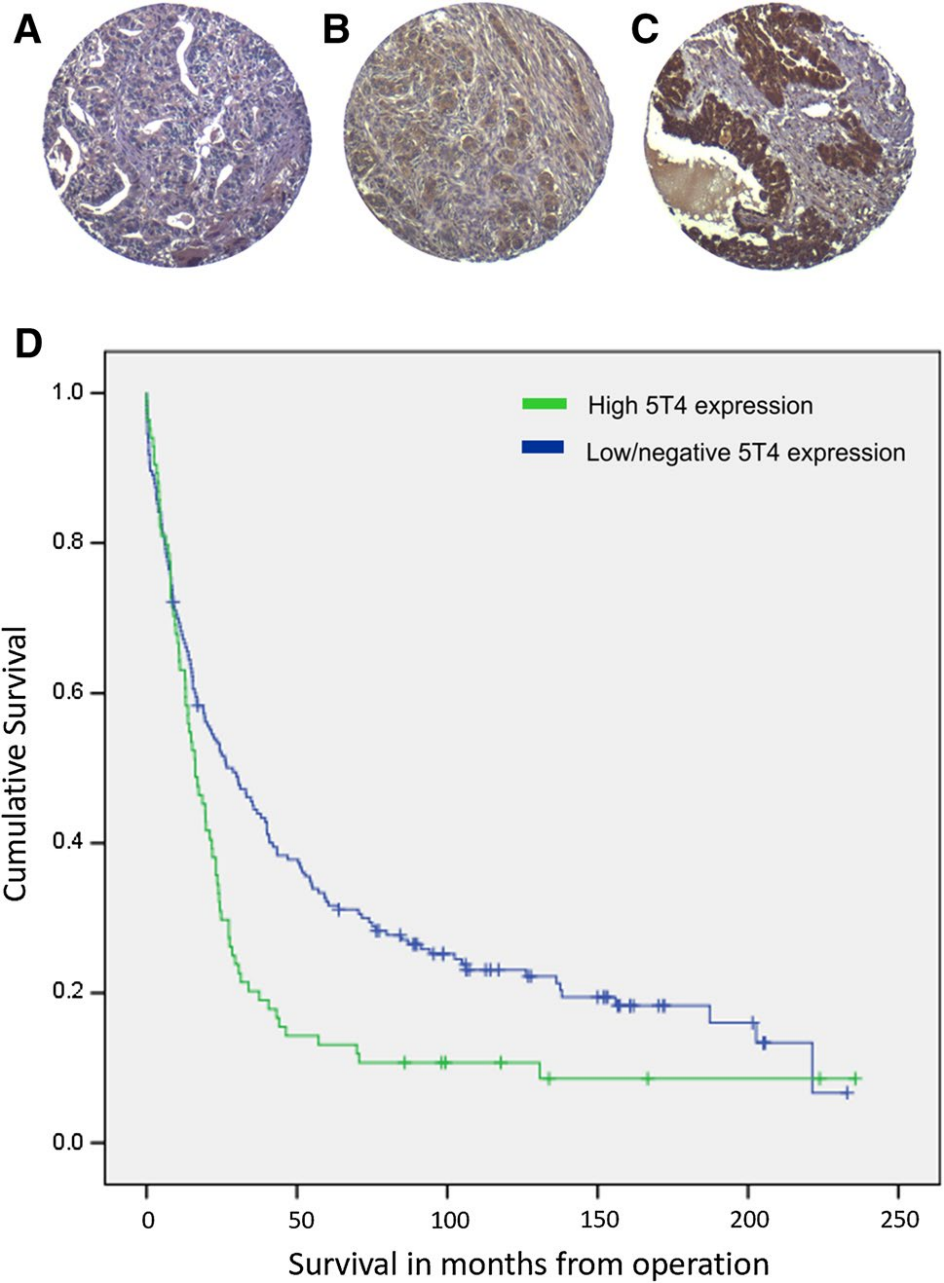
Immunohistochemical assessment of 5T4 expression in an ovarian cancer cohort. Representative photomicrographs of ovarian tissue microarray cores immunohistochemically stained for 5T4 are shown. The level of expression ranged from negative (**a**), weak (**b**) to strong expression (**c**). Kaplan–Meier survival curves of patients stratified by high and low 5T4 expression are shown in **d**

**Fig. 2:**
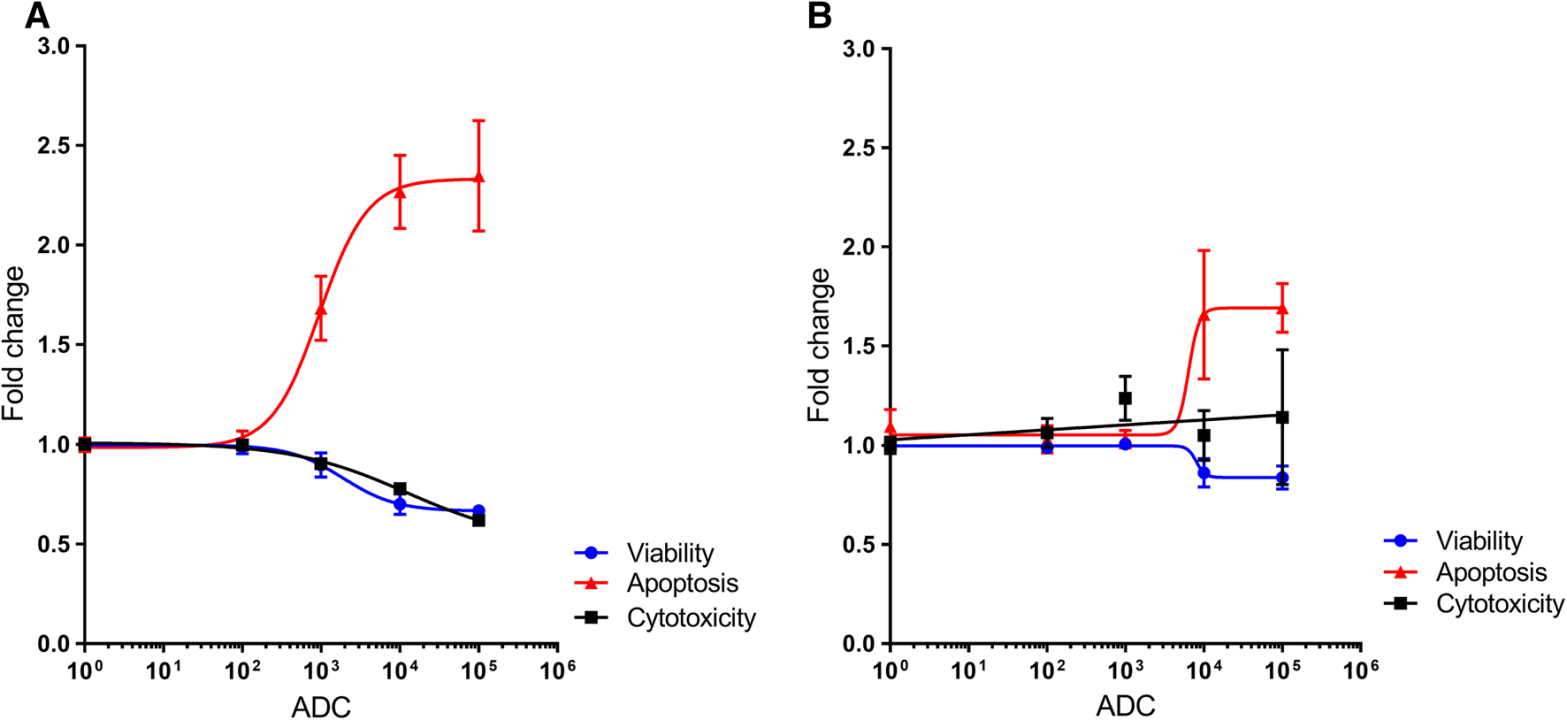
In vitro activity of A1mcMMAF. The 15 × 10^3^ cells were plated in triplicate/quadruplicate and treated with increasing doses of A1mcMMAF for 48 h. Apotox Glo assays were performed to assess live and dead cell protease activity and apoptosis concurrently. Batch normalised readings from three independent experiments (*n* = 10) are shown. **a** 5T4 wild-type cells and **b** 5T4 knockout cells. *ADC* antibody–drug conjugate

The SKOV3 cell line was obtained from ATCC and cell line authentication by a short tandem repeat analysis was performed. This cell line shows high levels of 5T4 cell surface expression [see Fig. 1 of the Electronic Supplementary Material (ESM)]. Cells were cultured at 37 °C, 5% CO_2_ in RPMI-1640 (Gibco, Grand Island, NY, USA) with 10% foetal bovine serum (Sigma-Aldrich, Irvine, UK) and 2 mM of l-glutamine (Gibco). SKOV3 cells were transfected, using Lipofectamine 3000 (Invitrogen, Carlsbad, CA, USA), with a pair of custom-designed TALEN vectors (Cellectis, Paris, France) targeting the start codon of the 5T4 gene. Eight days after transfection, fluorescence-activated cell sorting was performed to enrich for 5T4-negative cells. Following expansion and further cell sorting, a population of 5T4 knockout SKOV3 cells was established (Fig. 2a of the ESM) that demonstrated no detectable 5T4 expression by flow cytometry or western blotting on serial passages (Fig. 2b of the ESM).

### MTS Viability In Vitro Dose–Response Assays

The 2 × 10^3^ cells were seeded in each well of a 96-well plate in triplicate in normal growth media in three independent experiments. After 24 h, the media was replaced with 100 µL of media supplemented with various concentrations of A1mcMMAF (0–100 μg/mL) (Pfizer, Pearl River, NY, USA) or control mcMMAF (Neg-8-8-hG1mcMMAF, 0–100 μg/mL) (Pfizer). SKOV3 cells have a doubling time of approximately 24 h. Therefore, cell viability was measured 72 h (i.e. three doubling times) after the first exposure to the drug using a colorimetric tetrazolium (MTS) assay (Promega CellTiter 96 AQueous One; Promega, Madison, WI, USA). Absorbance at 495 nm was measured within 2 h of adding the MTS substrate.

### Multiplex Viability, Apoptosis and Necrosis In Vitro Dose–Response Assays

The 15 × 10^3^ cells were plated in triplicate/quadruplicate in 96-well, white, flat-bottom, cell culture-treated assay plates in three independent experiments. After 24 h, normal growth media was replaced with 100 μL of media supplemented with A1mcMMAF (10–100 μg/mL). Forty-eight hours after the first drug exposure, 20 μL of a viability/cytotoxicity substrate containing 10 μL of GF-AFC and 10 μL of bis-AAF-R110 were added to each well (ApoTox-Glo, Triplex Assay; Promega) to enable simultaneous measurement of each parameter with minimal degradation of proteases secreted at early time points. After 30 min at 37 °C, the fluorescence was measured using a Fluostar Optima plate reader (BMG Labtech, Ortenberg, Germany). To measure apoptosis, 100 μL of Caspase-Glo 3/7 reagent was added to each well and incubated at room temperature for 30 min (ApoTox-Glo, Triplex Assay; Promega). Luminescence was then measured using the Fluostar Optima plate reader.

### In Vitro Combination Viability Assays

Five hundred SKOV3 cells were seeded in each well of a 96-well plate in triplicate in three independent experiments. Twenty-four hours after seeding, culture media was changed for media supplemented with doses of carboplatin (Accord Healthcare, Middlesex, UK) ranging from 1 to 16 mg/mL, A1mcMMAF of 0.25–4 μg/mL or a combination as shown in Table 1 of the ESM. The culture media was changed at 72 h and fresh media containing the drugs was added. Viability was measured using the MTS assay as detailed above 120 h after the first exposure to the drug to capture later effects on viability as previously reported by Shor et al. [29]. The mean normalised fractional inhibition from three independent experiments was calculated; with a fractional inhibition of 1.0 equating to an absorbance reading in a blank well and 0 equating to an absorbance reading of the untreated cells. The concentration of the drug required to halve viability (50% inhibitory concentration) and the combination index was calculated using the Chou–Talalay method [35, 36] in Compusyn.

### In Vivo Antibody–Drug Conjugate Therapy

SKOV3 Lenti/Luc/Green Fluorescent Protein (GFP) were transduced with retroviral vector rKat co-expressing the *firefly* luciferase (Luc2) and GFP (kindly gifted by Dr. David Gilham, University of Manchester, UK) by centrifugation in viral supernatant at 1200 × *g* for 3–4 h in the presence of 4 μg/mL of polybrene (Sigma-Aldrich, St Louis, MO, USA). After 14 days of culture, Influx FACS sorting was used to enrich for GFP-positive cells and after further expansion and sorting a homogeneous SKOV3 Lenti/Luc/GFP ovarian cancer cell line was obtained. These cells (0.5–1 × 10^6^) were given intraperitoneally to NOD-*scid*IL2Rgamma^null^ (NSG) mice (Charles River, Harlow, UK) to model residual microscopic tumour load within the abdomen following primary surgical debulking. Mice were treated with either A1mcMMAF or control mcMMAF at a dose of 5–10 mg/kg intraperitoneally beginning 5 days after the tumour challenge with a cycle of three or four doses of ADC given at 4-day intervals (treatment block of 12–16 days). Where second courses were given, a rest period of 1 week was left between treatment blocks to mimic clinical dosing schedules. SKOV3 Lenti/Luc/GFP intraperitoneal challenge was monitored by IVIS (In vivo imaging system) [IVIS Lumina III, PerkinElmer, Waltham, MA, USA] as previously described [18]. Four weeks of weekly carboplatin at various doses was compared to combination carboplatin and A1mcMMAF in SKOV3 Lenti/Luc/GFP xenografts established in NSG mice. Efficacy was determined by time to exponential growth as determined by IVIS and survival.

### Statistical Analysis

Statistical analysis of the immunohistochemistry data was performed using SPSS20 statistical software (SPSS Inc., Chicago, IL, USA). Pearson’s *χ*^2^-tests and Fisher’s exact tests were used to determine the significance of associations between categorical variables. Survival rates were calculated using the Kaplan–Meier method; differences between survival curves were tested using the log-rank test. The Cox proportional-hazards model was used for multivariate analysis to calculate the hazard ratios. In all cases, two-sided *p*-values of < 0.05 were considered as statistically significant.

Statistical analysis of the cell model data is representative of three or more experiments, with the exception of long-term survival data. GraphPad Prism software (GraphPad Software, San Diego, CA, USA) was used for individual or multiple-group comparisons by a two-tailed Student *t* test or analysis of variance (Tukey’s test).

## Results

### Correlation of 5T4 Expression in Ovarian Cancer with Clinicopathological Variables and Overall Survival

The expression of 5T4 was assessed by immunohistochemistry using a tissue microarray comprising 360 primary ovarian cancers. In total, 271/360 ovarian cancer cores contained sufficient tumours for evaluation. The clinical characteristics of both the evaluated cohort and the original cohort are shown in Table 1. The clinical characteristics of the cohort evaluated for 5T4 expression was no different from that of the original cohort.

The results showed 38/271 (14%) negative tumours, and 145/271 (54%) and 87/271 (32%) tumours stained weakly or strongly, respectively for 5T4 (Fig. 1a–c). Cytoplasmic and membranous staining was seen predominantly in the epithelial component of the tumour cores. Stromal staining was seen in 2/89 (2%) cores that contained insufficient tumours for analysis.

Kaplan–Meier analysis showed a significant difference in survival for patients with 5T4 levels (high vs. low/negative expressing tumours) (log rank test, *p* = 0.003). Women with tumours showing no or low 5T4 expression showed a 75% increase in median survival from 16.1 months (95% confidence interval 11.0–21.2) in the 5T4 high group and 28.7 months (95% confidence interval 18.7–38.7) in the 5T4 low group (Fig. 1d).

Univariate analysis was performed to determine whether 5T4 expression correlated with standard clinicopathological variables (Table 2). Pearsons *χ*^2^ and Fisher’s exact tests indicated that high 5T4 expression did not correlate with other prognostic factors such as age (*p* = 0.792), International Federation of Gynecology and Obstetrics (FIGO) stage (*p* = 0.117), grade (*p* = 0.711), or macroscopic residual disease (*p* = 0.126). High 5T4 expression was, however, associated with histological type (*p* = 0.004); specifically, high-grade serous histology. These women were significantly more likely to progress to adjuvant therapy (*p* = 0.044).

**Table 2:** Univariate analysis of 5T4 expression in correlation with standard clinicopathological variables using the *χ*^2^- or Fisher’s exact test

Variable	*χ*^2^-test (*P* value)
	5T4
SEER age	0.792^a^
Tumour FIGO stage	0.117^a^
Tumour grade	0.711^a^
Macroscopic residual disease	0.126^a^
Adjuvant therapy	0.044
Histological type	0.004^a^
CXCL12	0.419

*FIGO* International Federation of Gynecology and Obstetrics, *SEER* Surveillance, Epidemiology, and End Results

^a^Pearsons Chi square test

*P* values < 0.05 are accepted to be significant

Univariate modelling determined that 5T4 expression (*p* = 0.006), CXCL12 expression [34] (*p* = 0.026), adjuvant therapy (*p* = 0.003), FIGO staging (*p* < 0.001), tumour grade (*p* = 0.001) and histotype (*p* < 0.001) were significantly associated with cancer-specific survival in this cohort (Table 3). After adjustment for the effect of these variables, 5T4 remained an independent prognostic factor (*p* = 0.032) (Table 4). Notably, FIGO stage (*p* < 0.001) and adjuvant therapy (*p* < 0.001), were also predictors of patient survival in this analysis, suggesting that 5T4 expression may be a useful prognostic marker in ovarian cancer.

**Table 3:** Univariate analysis of cancer-specific survival in women with epithelial ovarian cancer

Variable	Category	% Expression	Mean disease-specific survival (months)	95% CI (months)	*P* value
5T4	Low	68	67	5–79	0.006
	High	32	43	30–57	
CXCL12	Low	39	76	59–92	0.026
	Medium	41	63	47–79	
	High	20	36	24–48	
Adjuvant therapy	No	29	88	66–110	0.033
	Yes	71	50	42–59	
FIGO stage	1	27	130	110–149	< 0.001
	2	11	77	46–109	
	3	50	28	22–35	
	4	12	15	9–22	
Tumour grade	1	12	74	48–100	0.001
	2	21	82	60–104	
	3	67	48	38–59	
Tumour type in order of lethality	Borderline	5	104	67–141	< 0.001
	Clear cell	7	119	78–160	
	Mucinous	10	86	55–116	
	Endometrioid	12	96	61–131	
	Serous	50	38	30–46	
	Undifferentiated	16	38	22–56	

*CI* confidence interval, *FIGO* International Federation of Gynecology and Obstetrics

**Table 4:** Multivariate analysis of cancer-specific survival in women with epithelial ovarian cancer

	Exp(*B*)	95% CI for Exp(*B*)		*P* value
		Lower	Upper	
FIGO stage				
Stage 1	1			< 0.001
Stage 2	0.087	0.044	0.172	
Stage 3	0.529	0.281	0.993	
Stage 4	1.009	0.629	1.619	
Adjuvant therapy				
Yes	1			< 0.001
No	2.434	1.514	3.912	
Tumour grade				
1	1			0.144
2	0.607	0.368	1.001	
3	0.965	0.659	1.414	
Tumour type in order of lethality				
Borderline	1			0.621
Clear cell	0.583	0.157	2.168	
Mucinous	1.006	0.273	3.712	
Endometrioid	0.630	0.180	2.202	
Serous	0.613	0.189	1.993	
Undifferentiated	0.574	0.170	1.940	
CXCL12				
Low	1			0.063
Medium	0.631	0.429	0.928	
High	0.740	0.507	1.082	
5T4				
Low	1			0.032
High	0.705	0.512	0.970	

The analysis is based on the Cox multivariate regression model

*CI* confidence interval, *FIGO* International Federation of Gynecology and Obstetrics

*P* values < 0.05 are accepted to be significant

### In Vitro Activity of Antibody–Drug Conjugate vs. SKOV3 Cells

**Fig. 3:**
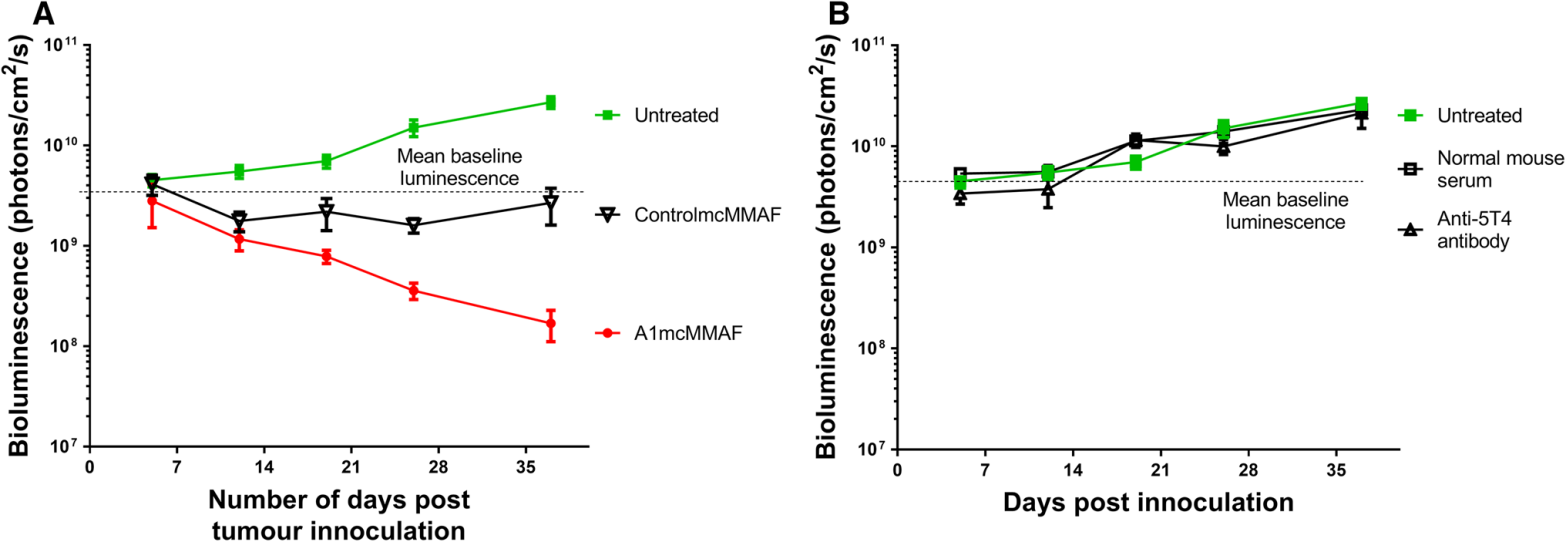
Drug conjugate confers cytotoxic effect seen in SKOV3 xenograft models. The 5 × 10^6^ SKOV3 Green Fluorescent Protein/luciferase cells were injected intraperitoneally into groups of six 8-week-old, NOD-*scid*IL2Rgamma^null^ mice. Mice were treated with a mouse monoclonal anti-human 5T4 antibody (H8) (5 mg/kg every 3 days for five doses), normal mouse serum (5 mg/kg every 3 days for five doses), A1mcMMAF (10 mg/kg every 4 days for three doses) or control mcMMAF (10 mg/kg every 4 days for three doses). Animals were imaged weekly. Mean bioluminescence for each group are shown

To model ADC activity in 5T4-expressing ovarian cancer cells, 5T4 knockout SKOV3 cell lines were created using 5T4-specific TALEN vectors (Fig. 2 of the ESM). 5T4-positive wild-type and 5T4 knockout SKOV3 cells were treated with increasing doses of A1mcMMAF for 72 h (i.e. three doubling times) and viability assessed by MTS assay. The 50% inhibitory concentration of A1mcMMAF in 5T4 knockout cells was 20-fold higher (1.1 μg/mL (95% confidence interval 0.7–2.0) vs. 21.4 μg/mL (95% confidence interval 14.2–32.5) (*p* < 0.0001) (Fig. 3 of the ESM).

**Fig. 4:**
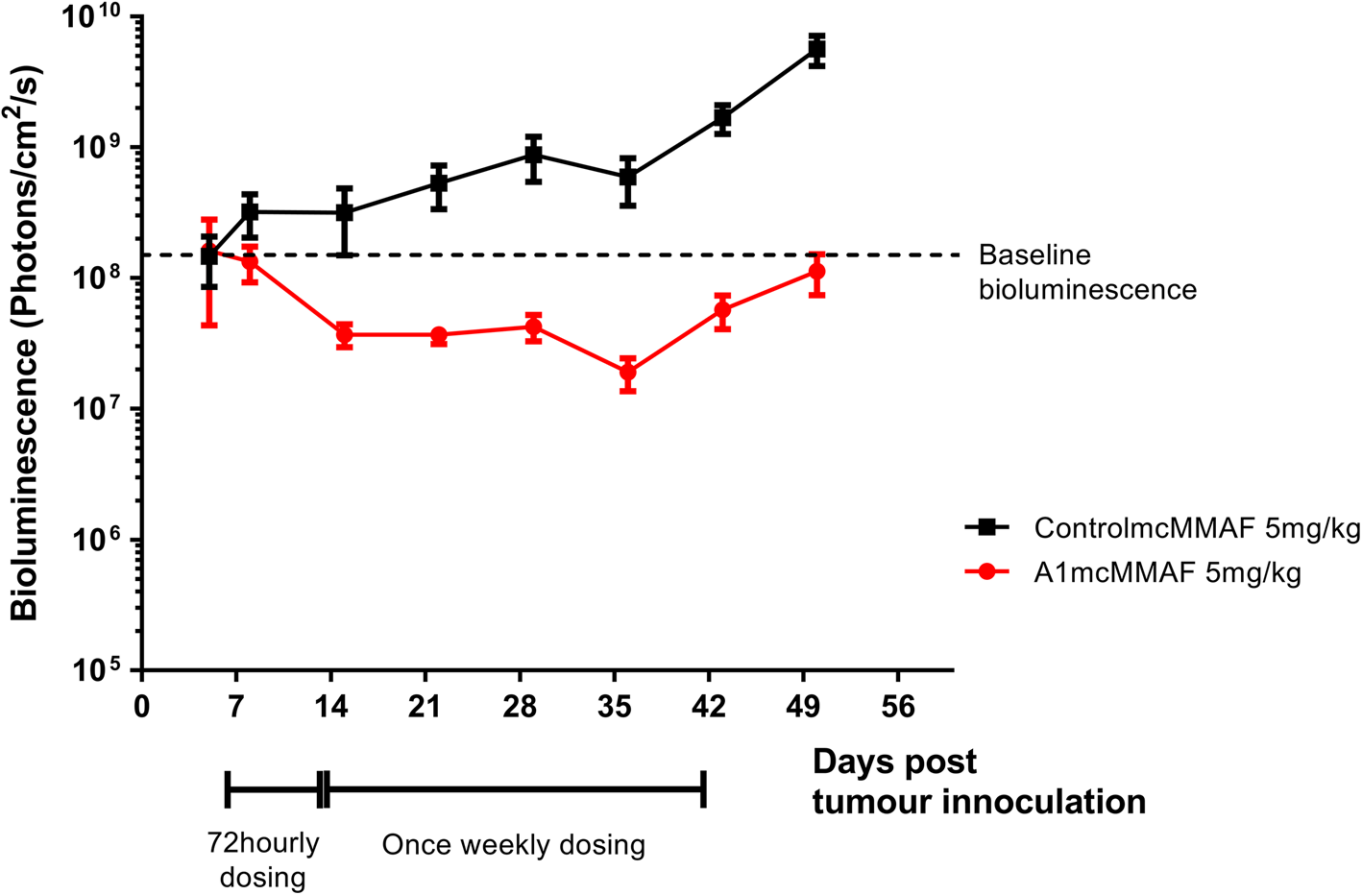
In vivo monotherapy with A1mcMMAF. The 5 × 10^4^ SKOV3 Green Fluorescent Protein/luciferase cells were injected intraperitoneally into groups of six 8-week-old, NOD-*scid*IL2Rgamma^null^ mice. Mice were treated with A1mcMMAF (5 mg/kg every 4 days for three doses) or control mcMMAF (5 mg/kg every 4 days for three doses) until day 13. Weekly doses of A1mcMMAF or control mcMMAF were then given until an increase in tumour load was seen. Animals were imaged weekly. Mean bioluminescence for each group are shown

A1mcMMAF was also compared to a non-specific control ADC (Neg-8-8-hG1mcMMAF) to investigate the requirement of specific binding for drug activity. The 50% inhibitory concentration in 5T4 wild-type SKOV3 cells was significantly lower with A1mcMMAF than the control drug (*p* < 0.001). This is consistent with the drug specificity being conferred by the 5T4 monoclonal antibody (Fig. 4a of the ESM). The control drug and A1mcMMAF demonstrate similar effects on the viability of knockout cells. This effect is more profound at higher concentrations, suggesting that at these doses there is likely to be some non-specific effect of A1mcMMAF.

The ApoTox-glo Triplex assay was used to simultaneously assess cell viability, apoptosis and necrosis to investigate the predominant mode of cell death caused by A1mcMMAF. As was seen in the MTS assays, viability falls with increasing doses of A1mcMMAF. There is a reciprocal increase in caspase 3/7 activity with the decreasing proportion of viable cells. This effect is much larger in SKOV3 wild-type cells (Fig. 2a) and occurs at lower concentrations of A1mcMMAF. Cytotoxicity is similar across this dose range in the knockout cells (Fig. 2b). In the SKOV3 wild-type cells, however, cytotoxicity appears to fall in a dose-dependent manner. The apparent lack of an inverse correlation between the ratio of viable to dead cells is likely owing to an underestimation of cytotoxicity resulting from degradation of dead cell proteases produced by early necrosis, rather than being an actual decrease in cytotoxicity.

### Effect of Drug vs. Non-Specific Antibody–Drug Conjugate In Vivo

To model whether A1mcMMAF might be effective as an adjuvant therapy in ovarian cancer, tumour cell line xenografts were generated in NSG mice. The tumour load in mice bearing SKOV3 tumours treated with A1mcMMAF or the control drug (10 mg/kg every 4 days for three doses) was compared to tumour loads in mice given no treatment. A1mcMMAF treatment led to a rapid decrease in tumour load compared with untreated mice, whilst those administered the control drug showed attenuation in the rate of growth of tumours (Fig. 3a). The non-specific effect seen with the control drug is consistent with that detected in vitro assays (Fig. 4a of the ESM). Tumour burden is unaffected by the monoclonal antibody without the cytotoxic payload, or normal mouse serum (Fig. 3b), suggesting that the effect on tumour burden is reliant upon the drug conjugate portion of the ADC.

To model maintenance chemotherapy following initial cytotoxic treatment, an independent cohort of xenograft mice was given an initial cycle (5 mg/kg every 4 days for three doses intraperitoneally) followed by weekly doses of A1mcMMAF or the control drug (5 mg/kg or equivalent) (Fig. 4). A1mcMMAF has been shown to accumulate in tumour cells with peak levels at 48 h post-dose, but significant amounts of drug remain detectable after 10 days [37]. Therefore, weekly maintenance doses were given with the aim of sustaining intra-tumoural drug concentrations sufficient to limit further tumour growth. Dosing at 5 mg/kg was used to reduce non-specific cytotoxicity.

**Fig. 5:**
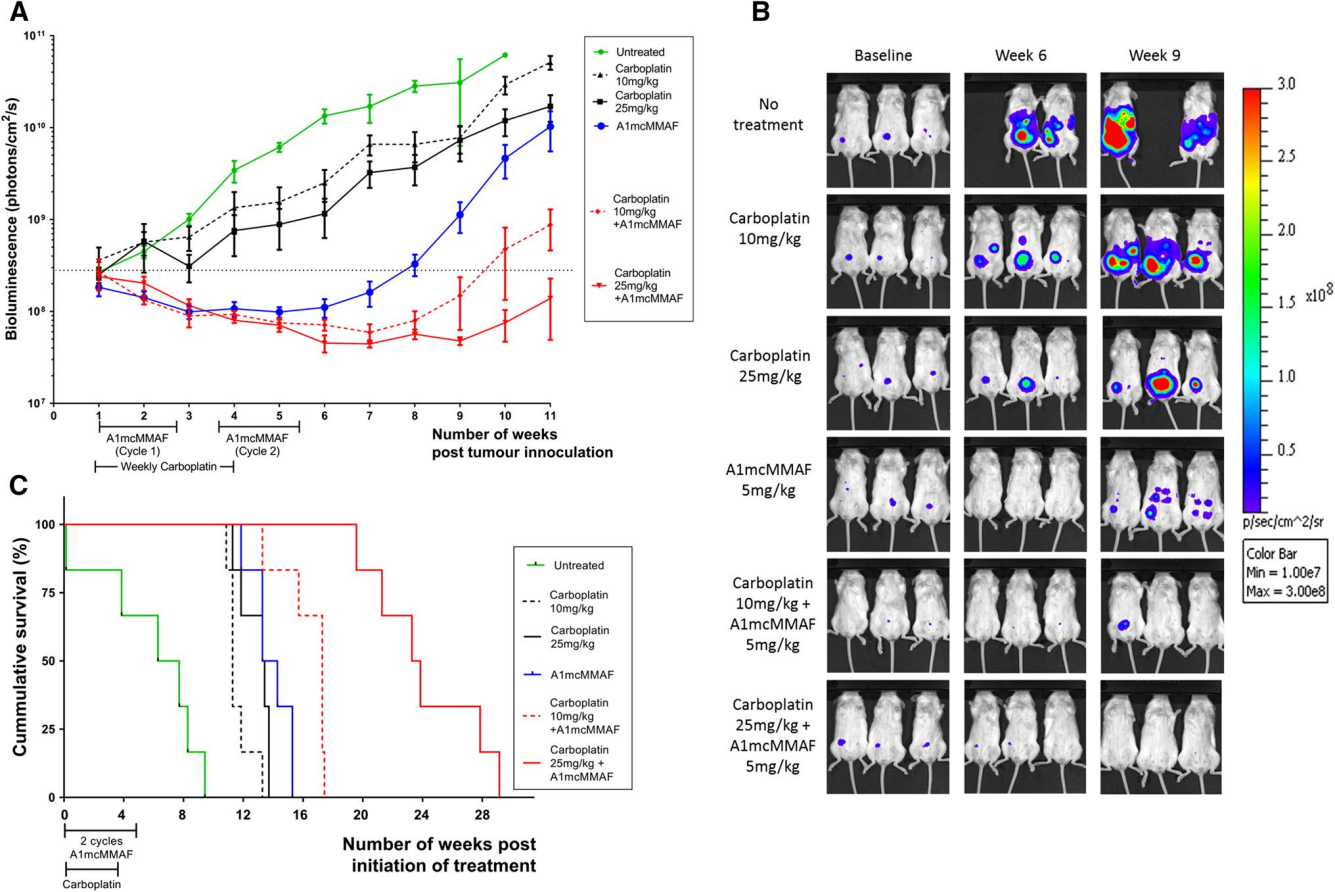
Effect of combining A1mcMMAF with carboplatin. The 1 × 10^5^ SKOV3 Green Fluorescent Protein/luciferase cells were injected intraperitoneally into groups of six 8-week-old, NOD-*scid*IL2Rgamma^null^ mice. Mice were treated with A1mcMMAF, carboplatin or a combination as outlined in Fig. 6 of the ESM. Animals were imaged weekly until progression was seen and followed clinically until the survival endpoint was reached. Mean bioluminescent signal for each group is shown in **a**. Bioluminescent overlay images for three animals from each group at baseline, on completion of treatment (week 6) and at relapse (week 9) showing anatomical distribution of recurrence and tumour load are shown in **b**. Overall survival curves for each group are shown in **c**. *Max* maximum, *Min* minimum

With A1mcMMAF treatment, there was an initial decrease in tumour load with the 4-day dosing regime. Tumour load was maintained at nidus levels for two doses whilst on weekly dosing but began to rise shortly before the third dose (i.e. after day 35) (Fig. 4). In contrast, tumour burden increases with treatment with the control ADC (Fig. 4). Tumours from the A1mcMMAF-treated animals continued to express 5T4 even when cytostasis was no longer maintained, suggesting that tumour escape is not caused by the outgrowth of 5T4-negative cells (Fig. 5 of the ESM).

### Effect of Combining A1mcMMAF with Carboplatin

The combination of carboplatin and A1mcMMAF was compared to single-agent treatment to examine antagonistic, additive or synergistic effects on cell death both in vitro and in vivo. In vitro, testing of combinations of carboplatin and A1mcMMAF combination showed no additional effect and in fact, predicted a moderately antagonistic effect (Table 1 of the ESM).

To test the ability of A1mcMMAF to work in combination with platinum-based chemotherapy in vivo, SKOV3 xenograft mice were given single-agent carboplatin, single-agent A1mcMMAF, combination therapy or no treatment as per the schedule in Fig. 6 of the ESM. The tumours in untreated mice grew exponentially after peritoneal seeding (Fig. 5a). Median time to five-fold change from baseline (Fig. 7 of the ESM) was 14 days. Tumour burden necessitated termination of all animals by day 66 (i.e. week 9/10) (Fig. 5c).

Treatment with carboplatin monotherapy (10 mg/kg and 25 mg/kg) attenuated tumour growth rates but tumour load did not fall below baseline with these regimes (Fig. 5a, b). Median time to a five-fold increase in tumour load was 23 days and 24 days, respectively, for low- and high-dose carboplatin monotherapy (Fig. 7 of the ESM). In contrast, mice given A1mcMMAF monotherapy showed a reduced tumour load by the end of the first cycle. This decline was not sustained during the second cycle of A1mcMMAF (Fig. 5a); however, tumour load was lower than baseline at the end of treatment (Fig. 5a, b).

Mice treated with a combination of A1mcMMAF and carboplatin showed a continued fall in tumour load into the second cycle of A1mcMMAF and a prolonged cytostatic effect following treatment cessation. At the end of treatment (week 6), there was no significant difference in the mean bioluminescence in the high- and low-dose carboplatin/A1mcMMAF combinations (*p* > 0.999, one-way analysis of variance) (Fig. 5a). The median time to an increase in tumour load was 72 days post-initiation of treatment in mice receiving the lower dose of carboplatin in combination with A1mcMMAF; a five-fold increase in the time to an increase in tumour load compared with untreated animals (*p* < 0.0001, Log-rank Mantel Cox test). At the end of the IVIS follow-up, the median time to a five-fold change had yet to be reached in the group of mice treated with A1mcMMAF in combination with higher dose carboplatin (Fig. 7 of the ESM). Trends in overall survival mirrored those of time to progression; with groups with the longest time to the five-fold increase showing the longest overall survival (Fig. 5c). No difference was seen in survival when comparing monotherapy with either agent (*p* = 0.15, Log-rank Mantel Cox test). Compared with A1mcMMAF treatment alone, the addition of 25 mg/kg of carboplatin increased median survival a further 71% (interquartile range 142–200 days) compared with A1mcMMAF alone (interquartile range 88–107 days). Overall, combination treatment increased median survival from 44 days in the untreated group to 96 days (i.e. 2.2-fold increase) and 163 days (i.e. 3.7-fold increase), respectively, in the low- and high-dose carboplatin combination regimes (*p* < 0.0001, Log-rank Mantel Cox test).

## Discussion

The effectiveness of ADCs depends on numerous factors including tumour expression levels of the target antigen, as well as the ability of tumour cells to internalise and cleave the conjugated drug and undergo apoptosis in response. More recently, it has been demonstrated that this activity can be further enhanced by concurrent administration of other chemotherapeutics [28, 29]. Our study confirms that 5T4 is expressed by all histotypes of EOC, but particularly in women with high-grade serous carcinoma and supports the findings demonstrated in other published series [11–13]. 5T4 cell membrane expression makes this an attractive target for antibody-delivered therapies, such as ADCs, that require binding to a cell surface marker and subsequent internalisation to deliver their cytotoxic payload intracellularly. In this cohort, 5T4 expression appears to be an independent poor prognostic factor for survival, even after accounting for tumour stage, histotype and adjuvant therapy. This is consistent with the hypothesis that 5T4 expression may have a functional role in relapse and/or metastasis. Selective elimination of tumour-initiating cells expressing 5T4 using ADCs was shown to be effective at prolonging survival in mouse models of both non small cell lung carcinoma (NSCLC) and pre-B acute lymphoblastic leukemia [26–28]. Demonstrating similar effects in EOC, a condition in which most women will relapse following their initial surgery and chemotherapy, could offer additional options for disease control. Conventional therapy tends to reduce tumour burden and improves symptoms but may fail to eradicate tumour-initiating cells, leading to eventual recurrence and drug resistance. Therapies targeting tumour-initiating cells may, therefore, work best alongside conventional therapies that debulk the tumour mass. Our intraperitoneally seeded xenograft mouse model sought to emulate the effect of adjuvant treatment regimes following complete surgical debulking.

We have demonstrated that the 5T4-targeting ADC, A1mcMMAF, has effect both in vivo and in vitro. Using paired wild-type and 5T4 knockout SKOV3 cells, we demonstrated that the specificity of A1mcMMAF is conferred by the antibody portion, whilst the anti-proliferative effect is conferred by the drug conjugate. At higher dose ranges, reductions in cell viability are seen in vitro and reductions in tumour loads are seen in vivo with the control ADC, suggesting that there is the potential for non-specific killing of non-antigen-bearing cells. This has been previously noted in the early preclinical development phase, where numbers of NSCLC-derived spheroids decreased when treated with the control ADC [26]. It is thought that this may result from phagocytosis of cleaved ADC produced by secreted tumour-associated proteases [26]. Reassuringly, toxicology studies in cynomolgus monkeys, which demonstrate comparable normal tissue staining patterns to humans, demonstrated that, neither the non-specific effect, nor the 5T4-specific effect of the ADC lead to any significant adverse events [26, 37–39]. Furthermore, phase I trials of the 5T4 ADC, PF-06263507, demonstrated a favourable safety profile for the drug [25].

Treatment with A1mcMMAF attenuates tumour growth and leads to dose-dependent reductions in tumour load. Previous work by Sapra et al. using various primary culture and cell line models suggest that the response to 5T4 ADCs is governed more by the presence of 5T4 expression, rather than antigen density, histological type or sensitivity to auristatin compounds [26]. Whilst we recognise that the demonstration of effect in a more high-grade serous cancer cell line such as OVSAHO or OVCAR 8 would add to the case for clinical trials of this treatment in patients with ovarian cancer, the evidence of effect in SKOV3 cells, which express high levels of 5T4, but are thought to represent a clear cell/endometrioid phenotype, support using 5T4 expression as an inclusion criterion in future trials, over histotype alone.

In our study, tumour loads increased rapidly following cessation of A1mcMMAF monotherapy (5 mg/kg). We have demonstrated that combination therapy with carboplatin, the first-line chemotherapeutic used in ovarian cancer, leads to a prolonged delay in tumour growth and an increase in overall survival. A potential explanation for the enhanced activity of MMAF in conjunction with carboplatin may lie in their distinct antineoplastic effects. MMAF inhibits cell division by inhibiting tubulin polymerisation, leading to cell-cycle arrest in mitotically active cells. Alkylating agents, such as carboplatin, damage DNA by the induction of DNA adducts and are effective in both actively dividing cells, as well as, the quiescent cells that are unaffected by A1mcMMAF. Synergy between platinum-based drugs and anti-mitotic agents has been proposed to occur because the cell-cycle arrest induced by antimitotic drugs hinders DNA repair and leads to the accumulation of toxic platinum–DNA adducts [40]. Synergism of 5T4 ADCs has been shown with other classes of drugs. Shor et al. demonstrate a superadditive effect when using A1mcMMAF with both taxanes and PI3 K/mTOR inhibitors preclinically [29]. They propose that the mechanism for the synergy between A1mcMMAF and taxanes is that, despite both being microtubule agents, the distinct sites of action of MMAF and taxol result in distinct effects on tubulin, which cooperate to modulate cell-cycle progression and microtubule dynamics. We have previously demonstrated that the combination of dexamethasone and A1mcMMAF is effective in B-ALL [28].

5T4 scores highly when assessed against predefined criteria for priority ranking target antigens for translational research [12, 41]. Its potential association with tumour progression, high expression levels in a wide range of different cancers, expression on tumour-initiating cells, cell surface location and immunogenicity have made it an equally attractive target for other immunotherapeutic modalities. Following promising results from phase I and II clinical trials of modified vaccinia Ankara-5T4 (Trovax^®^) [42–52], a viral vector designed to induce a 5T4-specific immune response, a phase II clinical trial of this drug was undertaken in relapsed ovarian cancer (TRIOC; NCT01556841). Results are awaited but a phase III trial of Trovax^®^ in metastatic renal cell carcinoma demonstrated that survival was improved in patients that mounted an antibody response [42]. Unfortunately, when all patients were considered no overall difference in survival was seen. Antibody–drug conjugates have the advantage that they do not rely on a patient’s ability to mount an immune response, making their activity more predictable in the short term. Like vaccine approaches, ADC efficacy may be limited by preformed antibodies. In the phase I trial of the 5T4 ADC, PF-06263507, 9% of patients exhibited anti-drug antibodies prior to treatment, rising to 17% of patients post-treatment [25]. Chimeric antigen receptor T cells provide a further alternative that avoids the requirement for patients to mount a specific immune response. Preclinical studies have demonstrated the feasibility of engineering 5T4-specific chimeric antigen receptor T cells that are active in ovarian cancer mouse models [13]. However, the chimeric antigen receptor T cell pipeline is extremely labour intensive and requires additional infrastructure, increasing potential costs for healthcare providers and patients when compared with ADCs. Antibody–drug conjugates, therefore, have the most potential for use in clinical practice.

## Conclusions

Antibody–drug conjugates are one of the most rapidly growing fields of immunotherapeutics with over 100 in different stages of development [53]. The potential of 5T4 ADCs to improve the effectiveness of platinum-based chemotherapy regimes in ovarian cancer offers an exciting opportunity to prolong progression-free survival and overall survival in women in both the primary treatment and relapse settings. The completion of the recent phase I dose-escalation study of A1mcMMAF in solid cancers [25] lays the foundation for further clinical trials to determine its efficacy in women with EOC.

## Electronic supplementary material

Below is the link to the electronic supplementary material.

Supplementary material 1 (PDF 937 kb)
