# Correction to: Combination Treatment with an Antibody–Drug Conjugate (A1mcMMAF) Targeting the Oncofetal Glycoprotein 5T4 and Carboplatin Improves Survival in a Xenograft Model of Ovarian Cancer

**DOI:** 10.1007/s11523-019-00677-x

**Published:** 2019-10-03

**Authors:** Y. Louise Wan, Puja Sapra, James Bolton, Jia Xin Chua, Lindy G. Durrant, Peter L. Stern

**Affiliations:** 1 Division of Cancer Sciences, School of Medical Sciences, Faculty of Biology, Medicine and Health, The University of Manchester, 5th Floor Research, St Mary’s Hospital, Oxford Road, Manchester, M13 9WL UK; 2 Oncology Research and Development, Pfizer Inc., 401 N. Middletown Road, Pearl River, NY 10954 USA; 3 Department of Histopathology, Manchester University NHS Foundation Trust, Oxford Road, Manchester, M13 9WL UK; 4 Academic Clinical Oncology, The University of Nottingham, City Hospital Campus, Hucknall Road, Nottingham, NG5 1PB UK; 5 Manchester Cancer Research Centre, Division of Cancer Sciences, School of Medical Sciences, Faculty of Biology, Medicine and Health, University of Manchester, Wilmslow Road, Manchester, M20 4BX UK

## Correction to: Targeted Oncology (2019) 14:465–477 10.1007/s11523-019-00650-8

The article Combination Treatment with an Antibody–Drug Conjugate (A1mcMMAF) Targeting the Oncofetal Glycoprotein 5T4 and Carboplatin Improves Survival in a Xenograft Model of Ovarian Cancer, written by Y. Louise Wan, Puja Sapra, James Bolton, Jia Xin Chua, Lindy G. Durrant and Peter L. Stern, was originally published under a CC BY-NC 4.0 license, but has now been made available under a CC BY 4.0 license. The article is forthwith distributed under a Creative Commons Attribution 4.0 International License (https://creativecommons.org/licenses/by/4.0/), which permits use, sharing, adaptation, distribution and reproduction in any medium or format, as long as you give appropriate credit to the original author(s) and the source, provide a link to the Creative Commons licence, and indicate if changes were made. The PDF and HTML versions of the paper have been modified accordingly.

The original article has been corrected.

**Open Access** This article is licensed under a Creative Commons Attribution 4.0 International License (https://creativecommons.org/licenses/by/4.0/), which permits use, sharing, adaptation, distribution and reproduction in any medium or format, as long as you give appropriate credit to the original author(s) and the source, provide a link to the Creative Commons licence, and indicate if changes were made.

