# Supplementary material for: Combination Treatment with an Antibody–Drug Conjugate (A1mcMMAF) Targeting the Oncofetal Glycoprotein 5T4 and Carboplatin Improves Survival in a Xenograft Model of Ovarian Cancer
Source: Target Oncol. 2019 Jul 22;14(4):465–77. doi: 10.1007/s11523-019-00650-8 (PMC6684567; doi:10.1007/s11523-019-00650-8)
Supplement: Supplementary file 1 — Supplementary material 1 (PDF 937 kb) [file 11523_2019_650_MOESM1_ESM.pdf]

Combination treatment with an antibody drug conjugate (A1mcMMAF) targeting the oncofetal glycoprotein 5T4 and carboplatin improves survival in a xenograft model of ovarian cancer

Y Louise Wan[1], Puja Sapra[2], James Bolton[3], Jia Xin Chua[4], Lindy G Durrant[4], Peter L Stern[5]

1. Division of Cancer Sciences, School of Medical Sciences, Faculty of Biology, Medicine and Health, The University of Manchester, 5th Floor Research, St Mary's Hospital, Oxford Road, Manchester, M13 9WL
2. Oncology Research & Development, Pfizer Inc., 401 N. Middletown Road, Pearl River, NY 10954, USA
3. Department of Histopathology, Manchester University NHS Foundation Trust, Oxford Road, Manchester, M13 9WL
4. The University of Nottingham, Academic Clinical Oncology, City Hospital Campus, Hucknall Road Nottingham NG5 1PB
5. Manchester Cancer Research Centre, Division of Cancer Sciences, School of Medical Sciences, Faculty of Biology, Medicine and Health, University of Manchester, Wilmslow Road, Manchester, M20 4BX

Running title: 5T4 ADC and carboplatin in an ovarian cancer xenograft model

Corresponding author: Peter L Stern

Mailing address: Manchester Cancer Research Centre, Division of Cancer Sciences, School of Medical Sciences, Faculty of Biology, Medicine and Health, University of Manchester, Wilmslow Road, Manchester, M20 4BX

Email: Peter.Stern@cruk.manchester.ac.uk

Supplementary Data

Supplementary Table 1: In vitro combination indices of A1mcMMAF and carboplatin in SKOV3 cells

Supplementary Figure 1: 5T4 expression levels in various ovarian cancer cell lines

Supplementary Figure 2: Generation and validation of 5T4 knockout SKOV3 cells

Supplementary Figure 3: Expression of 5T4 sensitises cells to A1mcMMAF

Supplementary Figure 4: The antibody portion (A1) of A1mcMMAF confers relative specificity for cells expressing 5T4

Supplementary Figure 5: Immunohistological 5T4 expression in tumours developing following treatment with A1mcMMAF

Supplementary Figure 6: Dosing schedule used in in vivo combinatory assay

Supplementary Figure 7:

	Drug concentration (mg/ml)		Fractional inhibition	IC50	R	Combination index
	Carboplatin	A1mcMMAF				
Carboplatin	16		0.996	2.0711	0.962	
	8		0.899			
	4		0.760			
	2		0.444			
	1		0.239			
A1mcMMAF		0.004	0.789	0.0013	0.973	
		0.002	0.633			
		0.001	0.430			
		0.0005	0.278			
		0.00025	0.051			
Carboplatin + A1mcMMAF (4000:1)	8	0.002	0.916	1.9076+0.0005	0.998	1.640
	4	0.001	0.779			1.419
	2	0.0005	0.554			1.190
	1	0.00025	0.233			1.226

IC50 - median effect dose, R -linear correlation coefficient of the median effect

Supplementary Table 1: In vitro combination indices

500 SKOV3 cells were seeded in each well of a 96 well plate in triplicate and treated with various doses of carboplatin, A1mcMMAF or a combination of the two 24 hours after seeding. After 120 hours, viability was measured using an MTS assay. The mean normalised fractional inhibition from 3 independent experiments is shown. A fractional inhibition of 1.0 equates to an absorbance reading of an empty well, whilst a fractional inhibition of 0.0 equates to the absorbance equivalent to untreated cells. Combination Index (CI) = 1 represents an additive effect, CI<1 represents a synergistic effect and CI>1 represents an antagonistic effect respectively.

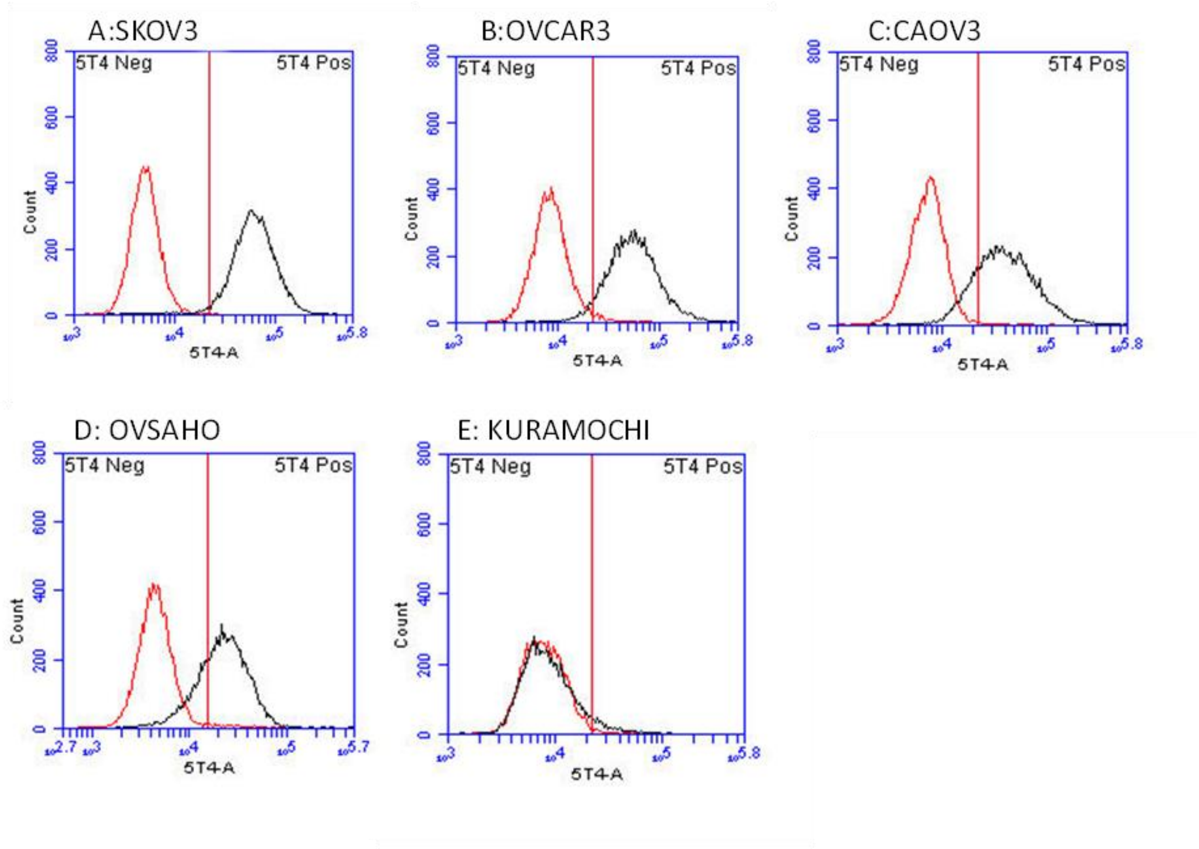

Supplementary Figure 1: 5T4 expression levels in various ovarian cancer cell lines

Cells from ovarian cancer cell lines were grown to confluency in normal growth conditions. 1x106 cells were labelled with 5T4mAb (shown in black) or IgG1 isotype control (shown in red) followed by an anti-mouse IgG1-AlexaFluor488 secondary at 1:1000. Representative histograms of fluorescence intensity of Alexafluor 488 are shown of the isotype control and 5T4 mAb labelled cells. (n>3)

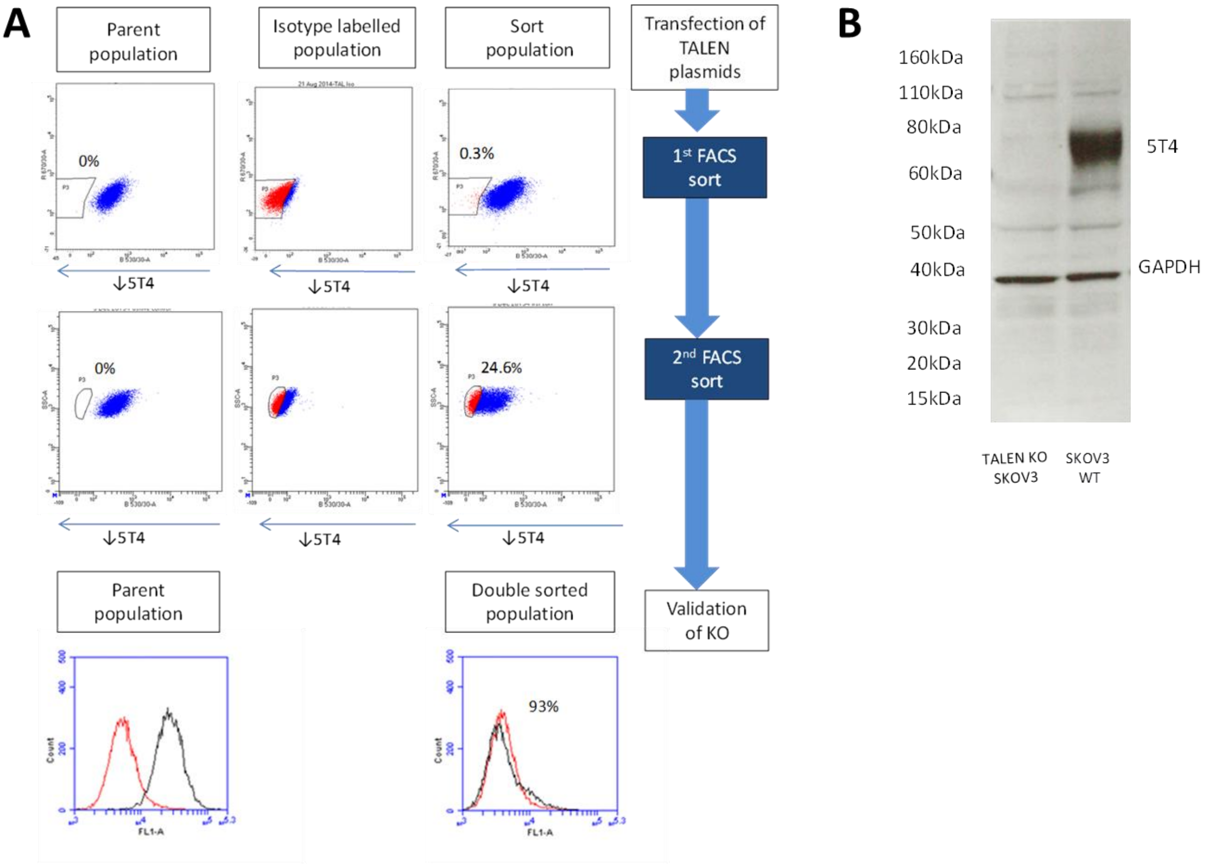

Supplementary Figure 2: Generation and validation of 5T4 knockout SKOV3 cells

A: 1.5x10^{6} WT SKOV3 cells were transfected with left and right TALEN knockout vector DNA. Day 8 after transfection these were labelled for 5T4 and sorted using FACS. Negative selection was used to select 5T4^{lo} cells (shown in red within the gate). 0.3% of the cells were selected in the first round of FACS sorting. Following enrichment, 24.6% of this population of cells was 5T4^{lo}. These cells form a polyclonal 5T4 knockout population where 93% of cells within this population have undetectable levels of 5T4 expression when validated by flow cytometry. Representative fluorescence intensity histograms are shown (Red – isotype control labelled, Black – 5T4AF488 labelled).

B: Reduced whole cell lysates (20µg) were subjected to 4-12% Bis-Tris SDS-PAGE prior to immunoblotting. A representative immunoblot of membranes labelled for detection of 5T4 followed by GAPDH is shown above. Unmodified populations demonstrate a wide band between 60-80kDa in keeping with the predicted molecular weight for the glycosylated version of 5T4, whilst the enriched polyclonal knockout cell lines show no bands at or around this molecular weight; confirming the absence of detectable 5T4.

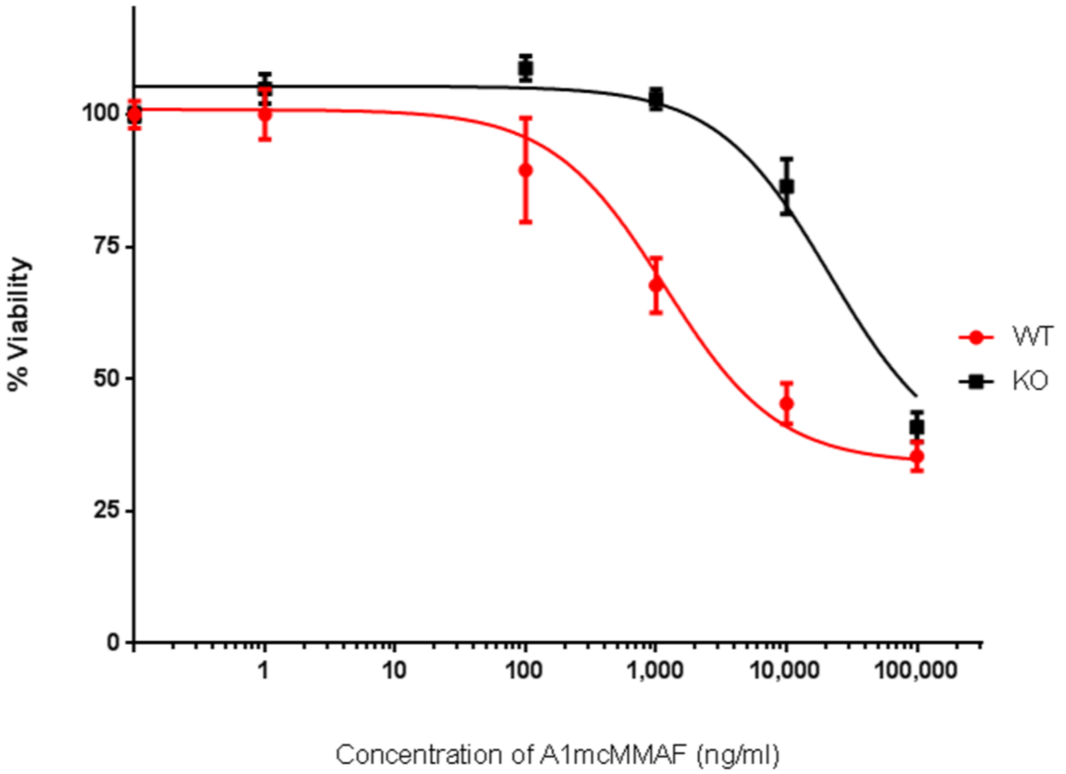

Supplementary Figure 3: Expression of 5T4 sensitises cells to A1mcMMAF

2x10^3 of either SKOV3 wildtype (WT) or 5T4 knockout SKOV3 (KO) cells were seeded in each well of a 96 well plate in triplicate and treated with increasing doses of A1mcMMAF next day for 72hrs. Viability was assessed using an MTS assay. Relative viability with increasing doses of A1mcMMAF in 3 independent experiments (n=9) is shown.

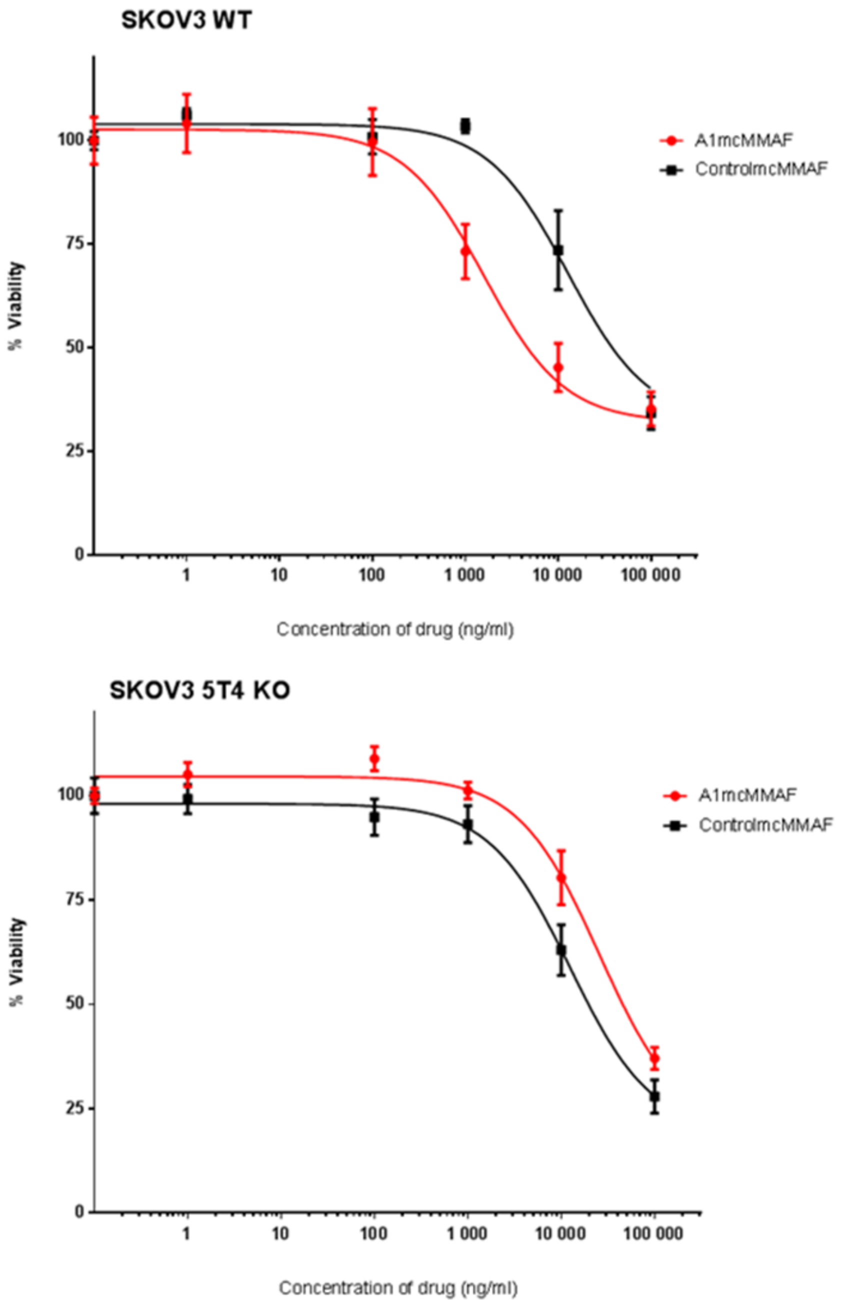

Supplementary Figure 4: The antibody portion (A1) of A1mcMMAF confers relative specificity for cells expressing 5T4

$2x10^3$ of either SKOV3 wildtype or 5T4 knockout SKOV3 (KO) cells were seeded in each well of a 96 well plate in triplicate and treated with increasing doses of A1mcMMAF or controlmcMMAF next day for 72hrs. Relative viability with A1mcMMAF and controlmcMMAF in 2 independent experiments (n=6) is shown.

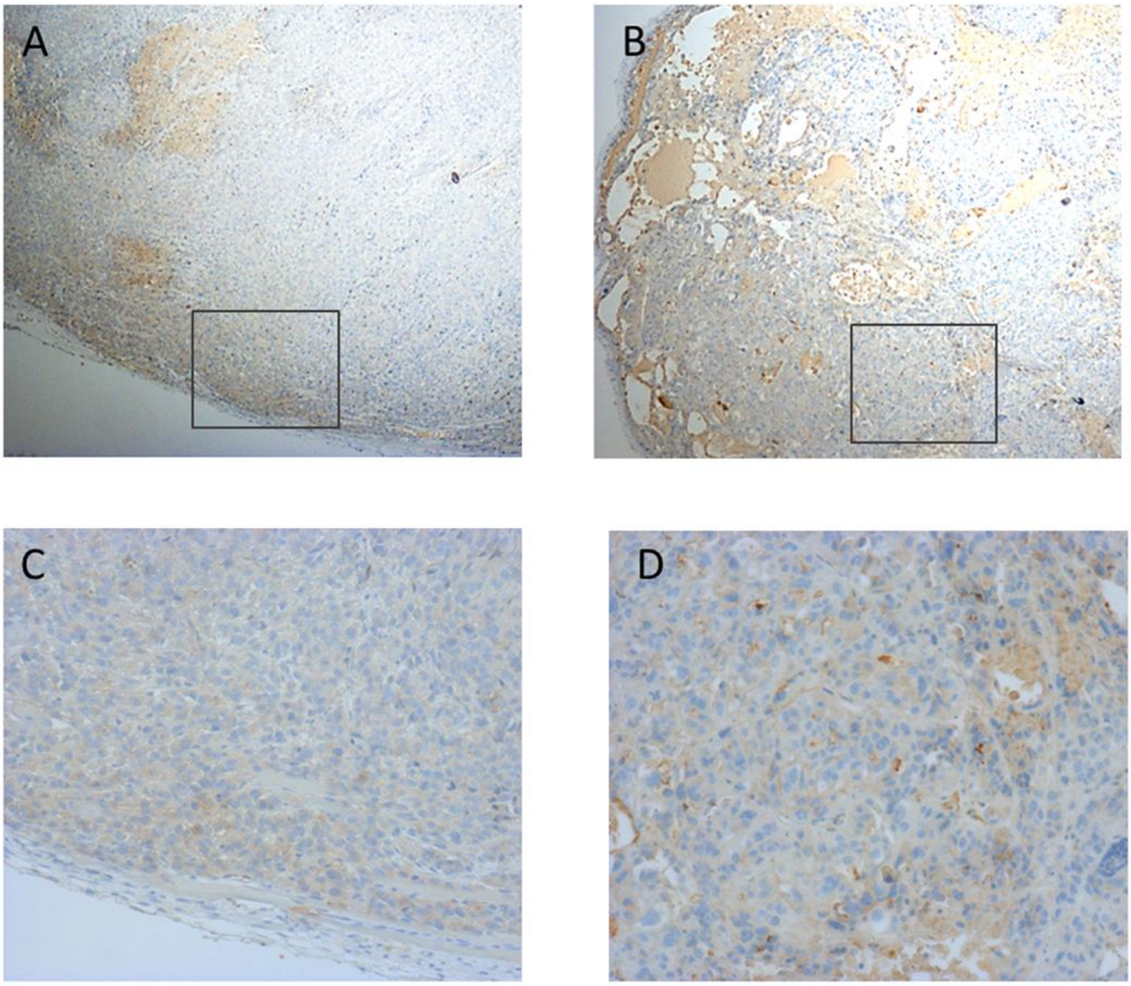

Supplementary Figure 5: Immunohistological 5T4 expression in tumours developing following treatment with A1mcMMAF

Tumour nodules were excised from untreated mice and mice displaying signs of relapse three weeks following cessation of A1mcMMAF treatment. 4µm sections were labelled for 5T4 (EPR 5229) at 1:1000 using a Ventana automated IHC system. (A and B – x20 magnification, C and D – x100 magnification). In untreated animals, heterogenous low level staining for 5T4 is seen (A). 5T4 expression is predominately seen in the periphery of the lesion (C). Following A1mcMMAF treatment and subsequent relapse, necrotic areas are seen interspersed with areas of low-level staining (B) similar to untreated animals (D).

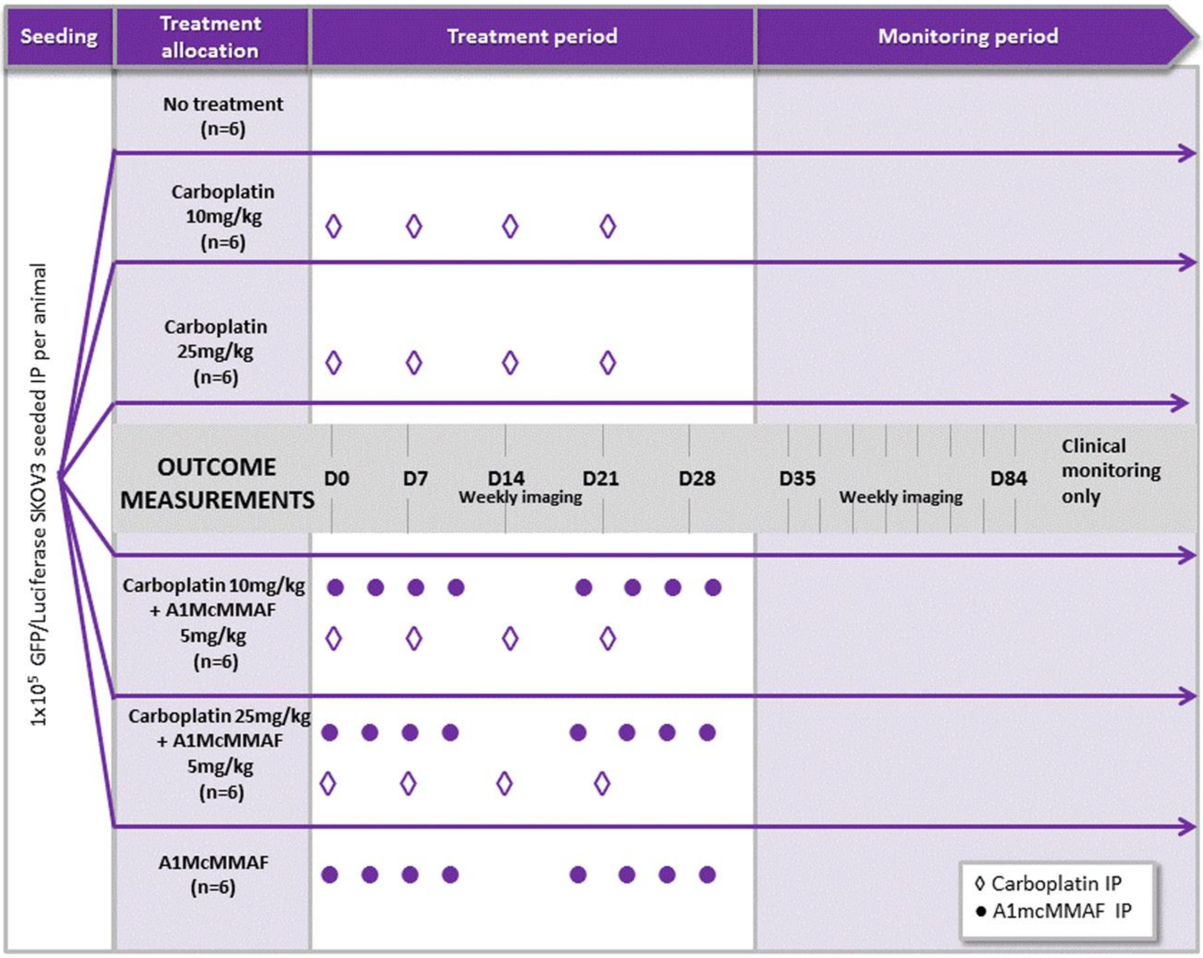

Supplementary Figure 6: Study flow chart showing dosing intervals and monitoring frequency used to investigate the effect of combining lower dose carboplatin with A1mcMMAF

Mice allocated to treatment arms receiving carboplatin will receive a total of either 2 mg (25 mg/kg) or 0.8mg (10 mg/kg) of carboplatin split over 4 doses. Mice receiving A1mcMMAF will receive a total of 800 µg of A1mcMMAF split over 8 doses. (Weight of average mouse =20 g). Those receiving a combination of the two doses received carboplatin 24 hours before receiving A1mcMMAF.

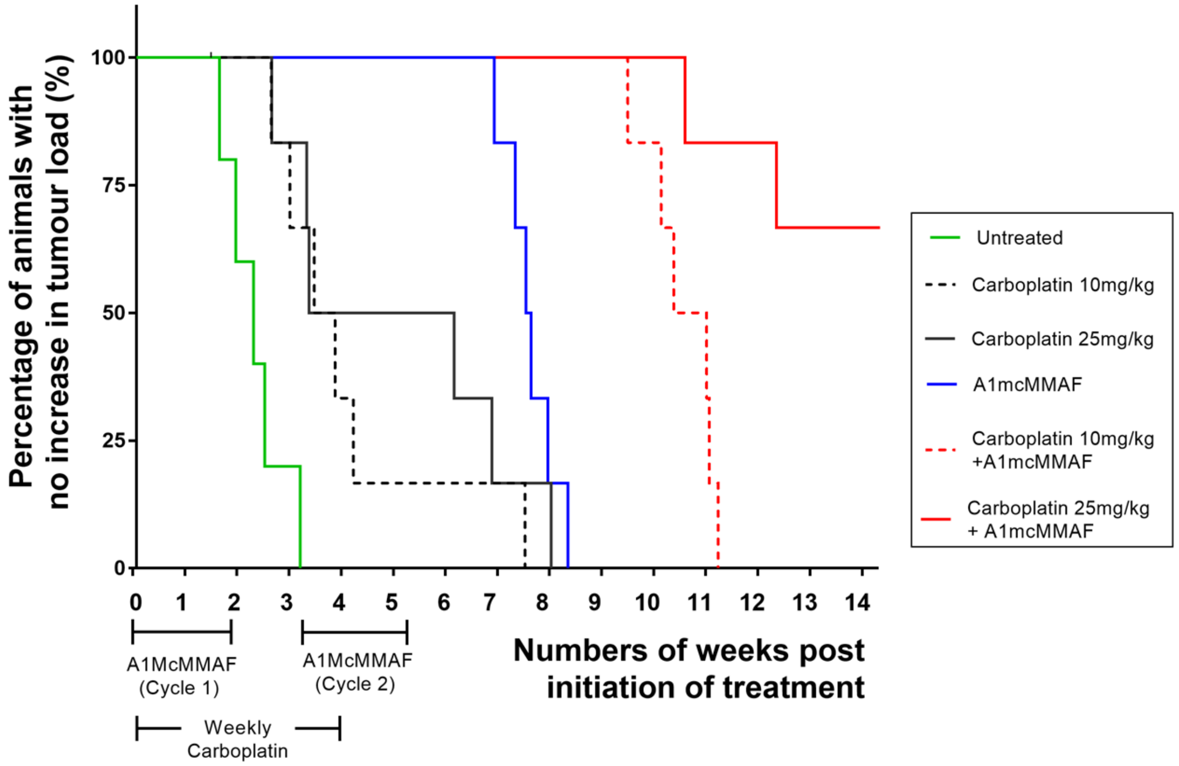

Supplementary Figure 7: Combination treatment with carboplatin and A1mcMMAF prolongs time to progression

Biomarker progression was determined by the time taken for there to be a five-fold increase in the tumour signal from baseline. Non-linear curve fitting was used to interpolate the time taken to reach this endpoint for each animal. Chemotherapeutic treatment with any agent significantly delayed time to five-fold increase in signal compared to untreated ($p<0.0001$, Log Mantel Cox). The addition of carboplatin at either dose to A1mcMMAF significantly delays increase in tumour load.
